# Potential Economic Viability of Two Proposed Rifapentine-Based Regimens for Treatment of Latent Tuberculosis Infection

**DOI:** 10.1371/journal.pone.0022276

**Published:** 2011-07-18

**Authors:** David P. Holland, Gillian D. Sanders, Carol D. Hamilton, Jason E. Stout

**Affiliations:** 1 Department of Medicine, Duke University Medical Center, Durham, North Carolina, United States of America; 2 Division of Infectious Diseases, Duke University Medical Center, Durham, North Carolina, United States of America; 3 Family Health International, Research Triangle Park, North Carolina, United States of America; Fundació Institut Germans Trias i Pujol; Universitat Autònoma de Barcelona CibeRES, Spain

## Abstract

**Rationale:**

Rifapentine-based regimens for treating latent tuberculosis infection (LTBI) are being considered for future clinical trials, but even if they prove effective, high drug costs may limit their economic viability.

**Objectives:**

To inform clinical trial design by estimating the potential costs and effectiveness of rifapentine-based regimens for treatment of latent tuberculosis infection (LTBI).

**Methods:**

We used a Markov model to estimate cost and societal benefits for three regimens for treating LTBI: Isoniazid/rifapentine daily for one month, isoniazid/rifapentine weekly for three months (self-administered and directly-observed), and isoniazid daily for nine months; a strategy of “no treatment” used for comparison. Costs, quality-adjusted life-years gained, and instances of active tuberculosis averted were calculated for all arms.

**Results:**

Both daily isoniazid/rifapentine for one month and weekly isoniazid/rifapentine for three months were less expensive and more effective than other strategies under a wide variety of clinically plausibly parameter estimates. Daily isoniazid/rifapentine for one month was the least expensive and most effective regimen.

**Conclusions:**

Daily isoniazid/rifapentine for one month and weekly isoniazid/rifapentine for three months should be studied in a large-scale clinical trial for efficacy. Because both regimens performed well even if their efficacy is somewhat reduced, study designers should consider relaxing non-inferiority boundaries.

## Introduction

Treatment of latent tuberculosis infection (LTBI) with isoniazid has long been established as an effective means to prevent the development of active tuberculosis [1], [2], [3] and is currently the standard of care in the United States and other high income countries [4], [5]. While such treatment clearly prevents TB-related morbidity and mortality, from a purely economic standpoint treating a case of LBTI is also less expensive than treating a case of active TB [6], [7], [8] and is therefore both economically and clinically desirable. Even accounting for the necessity of treating multiple individuals with LTBI to prevent one instance of active TB, currently-recommended regimens are expected to be cost-saving compared to a strategy of no treatment [6].

Despite its proven benefit, the overall utility of isoniazid monotherapy has been limited, as nearly half of patients started on isoniazid fail to complete a full course.[9], [10] Shorter regimens lead to improved completion rates [10], [11] but are not always cost-effective [7]. Therefore, it would be prudent to demonstrate the economic viability of any proposed regimen prior to testing it in a large-scale clinical trial.

Recently, new interest has focused on regimens containing rifamycins, particularly rifapentine, for LTBI treatment [12], [13]. A recently-completed large-scale clinical trial of isoniazid plus rifapentine given weekly for three months (the PREVENT TB study) found the efficacy of this shorter regimen to be non-inferior to isoniazid monotherapy but with much better completion rates [14]. However, in this study directly-observed therapy (DOT) was used to improve adherence, greatly increasing the regimen's cost. Another option, self-administered isoniazid plus rifapentine given daily for one month, has been proven efficacious in the murine model [15] and is currently being considered for study in patients with human immunodeficiency virus infection. Because this regimen is not intermittent, it could be given without DOT (current guidelines recommend DOT for all intermittent regimens [4]) and would be even shorter, perhaps increasing completion rates even further.

Of course, these advantages are currently only theoretical, and they come with a price. Daily rifapentine is relatively expensive, so any benefits from this new regimen would need to be sufficient to offset this higher cost. *Post hoc* cost-effectiveness analysis has traditionally been utilized for answering questions related to the economic viability of new interventions or strategies, but in an effort to increase the efficiency of clinical trial design, we propose a “*pre hoc*” cost-effectiveness analysis of two rifapentine-containing regimens to determine thresholds of key parameters that would determine the regimens' economic viability. Consideration of these thresholds can help study planners determine which treatment options have the greatest potential of economic viability and therefore should be of highest priority.

## Methods

We modified a previously-described Markov model created with TreeAge Pro 2009 (release 1.0.2; TreeAge Software, Inc., Williamstown, MA) to compare the costs, effectiveness, and cost-effectiveness of four different regimens for treating a cohort of individuals recently infected with TB:

1. Isoniazid 300 mg daily for 9 months, self-administered (9H-SAT daily, 270 doses)
2. Isoniazid 900 mg plus rifapentine 900 mg once-weekly for 3 months, self-administered (3HP-SAT weekly, 12 doses)
3. Isoniazid 900 mg plus rifapentine 900 mg once-weekly for 3 months, by directly-observed therapy (3HP-DOT weekly, 12 doses)
4. Isoniazid 300 mg plus rifapentine 600 mg daily for 1 month, self-administered (1HP-SAT daily, 30 doses).

Full details of the model (including schematic) are described elsewhere [6] but briefly, all individuals in the hypothetical cohort were assumed to start “on treatment.” Patients were moved to “off treatment” once they completed their regimen, experienced severe toxicity, or stopped due to non-adherence. All individuals were at risk of developing active TB, although that risk was decreased by treatment with each of the regimens; partial protection was afforded to patients who stopped treatment early in the 9H [16] or 3HP (assumed) arms (see Table 1). Persons who developed active TB were at risk of dying from TB during their treatment period, but their risk of death reverted to age-specific mortality once treatment was completed. We assumed that all individuals with active TB who did not die were successfully treated and did not relapse.

**Table 1:** Base-case parameters and probabilities used in the model.

Variable	Base-Case Estimate	Range	Reference
Lifetime probability of TB activation	0.06	0.06–0.4	[4], [17], [18], [19]
TB risk reduction from 9H-SAT daily:			
0–2 months	0		[16]
3–5 months	0.21	0.14–0.21	[16]
6–8 months	0.69	0.44–0.69	[16]
9 months	0.93	0.60–0.93	[16]
TB risk reduction from 3HP weekly (SAT or DOT):			
0–1 months	0		(assumed)
2 months	0.47		(interpolated)
3 months	0.93	0.60–0.93	[14]
TB risk reduction from 1HP-SAT daily:			
0 months	0		(assumed)
1 months	0.93	0.6–0.93	(assumed)
Probability of non-adherence (other than toxicity):			
9H-SAT daily	0.47	0–1	[9], [20]
3HP-DOT weekly	0.10	0–1	[14]
3HP-SAT weekly	0.13	0–1	[16], assumed
1HP-SAT weekly	0.05	0–1	(assumed)
Probability of severe toxicity (treatment stops):			
9H-SAT daily	0.014	0.001–0.2	[9], [14], [16]
3HP weekly (SAT or DOT)	0.05	0.006–0.03	[14]
1HP-SAT daily	0.02	0.005–0.10	(assumed)
Probability of hospitalization after severe toxicity	0.015	0.01–0.02	[21]
Probability of death due to drug toxicity	0.003	0–0.01	[16], [21]
Probability of extended treatment (active disease)	0.124		[22]
Probability of death from TB	0.04	0.03–0.05	[23]
Number of secondary cases per active case	1.2	0–1.2	[16], [24]

*9H = isoniazid daily for 9 months, 3HP = isoniazid plus rifapentine weekly for 3 months, 1HP = isoniazid plus rifapentine daily for 1 month. SAT = self-administered therapy, DOT = directly-observed therapy.*

Costs were updated to 2011 U.S. dollars, and efficacy, toxicity, and adherence parameters for 3HP-DOT weekly were updated based on the recently-completed clinical trial [14]. Base-case parameter estimates are shown in Table 1, utility adjustments are shown in Table 2, and base-case estimates for costs (U.S.) are shown in Tables 3 and 4.

**Table 2:** Utility adjustments for events occurring in the model, expressed as fractions of a life-year.

Event	Adjustment	Range	Reference
LTBI treatment	0.97	0.95–0.97	[25]
Treatment-limiting toxicity	0.75	0.65–085	(assumed)
Hospitalization	0.50	0.40–0.60	(assumed)
Treatment of active TB	0.90	0.64–0.93	[25]
Prior TB	0.95	0.85–1	(assumed)

**Table 3:** Costs in US$ associated with treating latent TB infection.

	Estimate	Range	Reference
9H-SAT daily cost per month:			
Number of doses	30		
Medications	$1.20		[26]
Monthly visit*	$26.52		[8]
DOT	$0		
Total	$27.72	$20–34	
3HP-DOT weekly cost per month	$174.62	$131–218	
Number of doses	4		
Medications	$53.68		[26]
Monthly visit*	$26.52		[8]
DOT	$96.30		[27], [28]
Total	$176.50	$133–221	
3HP-SAT weekly cost per month:			
Number of doses	4		
Medications	53.68		[26]
Monthly visit*	$26.52		[8]
DOT	$0		
Total	$80.20	$60–100	
1HP-SAT daily cost per month:			
Number of doses	30		
Medications	$267.30		[26]
Monthly visit*	$26.52		[8]
DOT	$0		
Total	$293.81	$220–367	
Severe toxicity costs:			
Lab monitoring (4 @$41.20)	$164.80	$124–206	[8]
Hospitalization (7 days)	$5,537.84	$4,153–$6,922	[29]

* *Average cost of routine monitoring and evaluation for mild toxicity under the assumption that 40% of individuals will require monthly monitoring of transaminases and 1.4% will have toxicity that will require a physician visit but not result in treatment discontinuation.*

**Table 4:** Costs in US$ associated with treating active TB.

	Total cost	Range	Reference
Diagnosis	$466.01	$350–583	[27]
Inpatient treatment	$10,402.37	$7,802–13,003	[27]
Outpatient (months 1 & 2)	$299.25	$224–374	[27]
Outpatient treatment (months 3+)	$261.01	$196–326	[27]
Contact tracing/testing	$488.65	$366–611	[28], [30]
Total per case - 6 months	$13,000		
Total per case - 9 months	$13,783		

Sensitivity analyses focused on parameters of the two trial regimens (daily isoniazid/rifapentine for one month and weekly isoniazid/rifapentine for 3 months) in an effort to determine threshold values above/below which these combinations would no longer be economically viable when compared to standard therapy (nine months of isoniazid) or to each other. Ranges for parameter estimates were taken from the available literature (U.S.) where available; where no literature was available, ranges were assumed as an approximation based on clinical judgment. Ranges for costs were determined by adding and subtracting 25% to the base-case estimate.

The model was run with cycles of one month duration over the life of each patient, and cohort analysis was used to calculate costs, quality-adjusted life-years (QALYs), and number of active cases. We followed recommendations from the Panel on Cost Effectiveness in Health and Medicine as appropriate [31]. Because we were interested in cost-saving regimens, a willingness-to-pay threshold of $0 was selected; therefore, in our analysis only regimens that were both more effective and less expensive than the standard of care (“dominant”) were considered a good use of resources.

## Results

**Figure 1:**
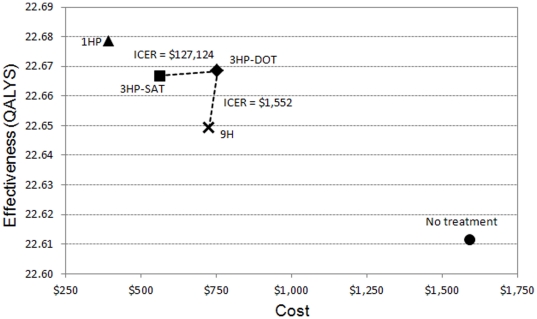
Cost-effectiveness plot of the four regimens and the “no treatment” strategy. Incremental cost-effectiveness ratios (ICER) are represented by the inverse slope of the dotted and dashed lines between strategies. *Abbreviations: 9H = isoniazid daily for 9 months, 3HP = isoniazid plus rifapentine weekly for 3 months, 1HP = isoniazid plus rifapentine daily for 1 month. SAT = self-administered therapy, DOT = directly-observed therapy*.

The base-case costs, QALYs, and incremental cost-effectiveness of the evaluated strategies are shown in Table 5 and Figure 1. All drug regimens dominated the “no treatment” strategy. The other regimens are summarized as follows:

1. 1HP-SAT daily dominated all other drug regimens
2. 3HP-SAT dominated 9H-SAT daily
3. 3HP-DOT weekly was more effective than 9H-SAT daily at a cost of $1,415 per QALY.

**Table 5:** Costs, effectiveness, and incremental cost-effectiveness ratios for the four drug regimens in order of increasing effectiveness, referenced to the strategy of “no treatment.”

Regimen	Cost per contact	Incremental cost	Effectiveness (QALYS)	Incremental effectiveness (QALYS)	Incremental cost-effectiveness ratio ($ per QALYS)	Cases of active TB per 1000 contacts
No treatment	$1,589	(ref)	22.61149	(ref)	(Dominated)	64
9H	$724	−$865	22.64937	0.037884	(Dominated)	22
3HP-SAT	$562	−$162	22.66685	0.017472	(Dominated)	15
3HP-DOT	$754	$192	22.66836	0.001511	(Dominated)	13
1HP	$392	−$362	22.67849	0.010128		10

### Sensitivity analysis

Pairwise comparisons were made between trial regimens and established regimens, and thresholds were calculated for key parameters above/below which the trial regimens were no longer cost-saving.

#### Adherence

If the adherence for 1HP-SAT daily is below 83% (base-case estimate = 95%), that regimen no longer dominates 3HP-SAT weekly; if its adherence is less than 71%, it no longer dominates 9H-SAT daily. If the adherence for 3HP-SAT weekly is below 70% (base-case estimate = 87%), it no longer dominates 9H-SAT daily, and below 67% it no longer dominates 3HP-DOT weekly.

#### Efficacy

Assuming base-case values for other parameters, the efficacy of 1HP-SAT daily could be as low as 81% (base-case estimate = 87%) and it would still dominate all other regimens; its efficacy could be as low as 70% and still dominate 9H-SAT daily.

#### Toxicity

If the rate of severe toxicity for 1HP-SAT daily is above 7% (base-case estimate = 2%), 3HP-SAT becomes the preferred regimen, though 1HP-SAT daily continues to dominate 9H-SAT daily until its rate of severe toxicity exceeds 10%. There were no thresholds for toxicity of 3HP within the specified sensitivity analysis range (0.6%–3%).

**Figure 2:**
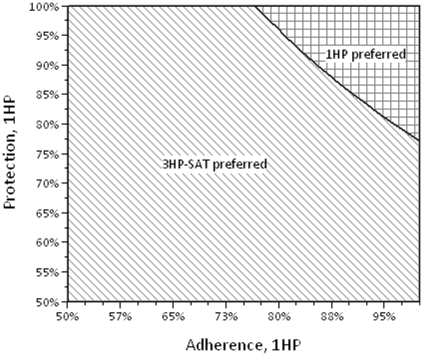
Two-way sensitivity analysis strategy graph comparing risk reduction and adherence for isoniazid/rifapentine daily for one month (1HP). The clear area shows combinations of adherence and risk reduction for 1HP that are high enough that 1HP is a cost-saving regimen. In the cross-hatched area, all combinations of adherence and risk reduction for 1HP are too low, so isoniazid/rifapentine monthly for 12 weeks self-administered (3HP-SAT) is preferred regimen.

**Figure 3:**
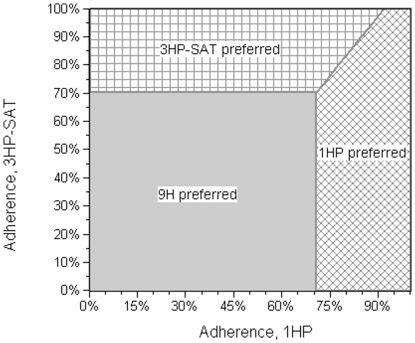
Two-way sensitivity analysis strategy graph comparing adherence for isoniazid/rifapentine daily for one month (1HP) vs. isoniazid/rifapentine weekly for three months self-administered (3HP-SAT). In the diagonal cross-hatched area, 1HP is cost-saving and therefore the preferred regimen. In the horizontal cross-hatched area, 3HP-SAT is cost-saving. In the shaded area, neither regimen is cost-saving when compared to isoniazid monotherapy daily for nine months (9H), which is the preferred regimen.

A two-way sensitivity analysis was done to assess the tradeoff between adherence and efficacy of 1HP-SAT daily and is shown in Figure 2. Also, under the assumption that all regimens are equally efficacious, a two-way sensitivity analysis was performed to show the effect of various adherence adjustments to the trial regimens 1HP-SAT daily and 3HP-SAT weekly; the strategy graph is shown in Figure 3.

Varying the model's costs and utilities in one-way sensitivity analyses over the range of estimates did not identify any thresholds where the recommended therapy would change. Likewise, varying the number of secondary cases per active case over the specified range did not identify any thresholds.

## Discussion

In our model, 1HP-SAT daily (isoniazid plus rifapentine self-administered daily for one month) was cost-saving compared to other options under a wide range of clinically plausible scenarios. Moreover, assuming high rates of adherence to this regimen, even if it was only 81% efficacious (compared to 93% for 9H-SAT daily) it would still maintain its overall economic advantage. 3HP-SAT weekly (Isoniazid plus rifapentine self-administered once-weekly for three months) also performed well compared to established regimens.

These results have important implications for proposed future clinical trials. First, they suggest that trials of 1HP-SAT daily and 3HP-SAT weekly are warranted, as successful demonstration of the efficacy of either proposed regimen would produce an option for treating LTBI that would be cost-saving compared to currently-available regimens. Second, the results indicate that tight non-inferiority bounds for efficacy would not be necessary, allowing for reduced sample size and a less expensive study.

A significant limitation of our study is that the point estimates for several parameters related to the proposed regimens are assumed. However, we chose these values merely as starting points for our analysis; the primary goal of our trial was to determine thresholds for these parameters that would make the regimens favorable or not favorable. Because our results suggest that the proposed regimens are economically advantageous over a wide range of estimates, we believe that any trial that would demonstrate the efficacy of these regimens would likely show adherence and toxicity values within the acceptable range shown in our study.

Another limitation of our study is that it is based on U.S. data and is therefore applicable only to similar settings. How these regimens would perform in areas of the world with high rates of reinfection is unknown. Also, we assumed the ability to accurately exclude active TB among members of the cohort. In areas of the world where diagnostic capability is somewhat limited, there may be an increased risk of drug resistance (possibly rifamycin resistance) among patients who develop active disease, which could dramatically alter costs. Further studies of these regimens in other areas of the world would be warranted.

We used drug costs from 2011 U.S. public health pricing. While drug prices are fluid, they tend to trend down over time. Because isoniazid is already very inexpensive, the primary driver of the cost difference among regimens is rifapentine; if it becomes cheaper, our results would only become more robust.

It is possible that TB reactivation rates in the contemporary era within the United States are significantly less than the older estimates (from 1975) used in our model [32]. However, even with a reactivation rate of half of the base-case estimate (6% lifetime risk), 1HP-SAT daily and 3HP-SAT weekly continued to outperform other options (including the “no-treatment” strategy). Likewise, with a relative risk of reactivation of 10 (corresponding to rates seen in untreated HIV infection [18]), these two regimens were still cost-saving compared to standard therapy. With activation rates as low as 0.004/year (estimated for low-risk reactors [32]), the “no treatment” regimen dominates other regimens except 1HP-SAT daily, which would still be considered cost-effective (incremental cost-effectiveness ratio $12,668) under most commonly-accepted willingness-to-pay thresholds for the U.S.

In summary, we have shown that treatment of LTBI with isoniazid plus rifapentine given either daily for one month or weekly for three months, all by self-administered therapy, has the potential to be cost-saving compared to standard therapy with isoniazid. We suggest that these two regimens should be studied in a randomized, controlled trial.
